# Paravertebral compartment syndrome after training causing severe back pain in an amateur rugby player: report of a rare case and review of the literature

**DOI:** 10.1186/1471-2474-14-259

**Published:** 2013-09-02

**Authors:** Georg Mattiassich, Lorenz Larcher, Markus Leitinger, Eugen Trinka, Gottfried Wechselberger, Heinrich Schubert

**Affiliations:** 1 Trauma Center Unfallkrankenhaus Linz, Teaching Hospital of the Paracelsus Medical University Salzburg, Linz, Austria; 2 Department of Plastic and Reconstructive Surgery, St. John of God Hospital, Teaching Hospital of the Paracelsus Medical University Salzburg, Linz, Austria; 3 Department of Neurology, Christian-Doppler-Klinik, Paracelsus Medical University and Salzburger Landeskliniken, Salzburg, Austria

**Keywords:** Paravertebral compartment syndrome, Back pain, Fasciotomy, Lumbar spine, Lumbago

## Abstract

**Background:**

Acute compartment syndrome (CS) of the paravertebral muscles without external trauma is rarely reported in literature. Not all of clinical symptoms for CS are applicable to the paravertebral region.

**Case presentation:**

A 30-year-old amateur rugby player was suffering from increasing back pain following exertional training specially targeting back muscles. He presented with hardly treatable pain of the lumbar spine, dysaesthesia of the left paravertebral lumbar region as well as elevated muscle enzymes. Magnetic resonance imaging (MRI) showed an edema of the paravertebral muscles. Compartment pressure measurement revealed increased values of 47 mmHg on the left side. Seventy-two hours after onset of back pain a fasciotomy of the superficial thoracolumbar fascia was performed. Immediately postoperatively the clinical condition improved and enzyme levels significantly decreased. The patient started with light training exercises 3 weeks after the operation.

**Conclusions:**

We present a rare case of an exercise-induced compartment syndrome of the paravertebral muscles and set it in the context of existing literature comparing various treatment options and outcomes. Where there is evidence of paravertebral compartment syndrome we recommend immediate fasciotomy to prevent rhabdomyolysis and further consequential diseases.

## Background

Compartment syndrome (CS) was initially described by Volkmann in 1881, and can have various etiologies. Pathophysiologically, CS is a multifactorial condition caused by increased tissue pressure in a closed fibro-osseous space, which may develop after trauma in the presence of certain cofactors. The local microcirculation is altered because of endothelial destruction, capillary leak, protein loss, and fluid accumulation in interstitial and intracellular third spaces [1].

CS of the extremities presents with the classical symptom of severe intractable pain, typically not relieved by non-narcotic pain medication, and the classical signs of pain on passive stretch, pallor, paresis and paresthesiae. These symptoms are not all applicable to the paravertebral region. Patients with CS may be at risk of multi-organ failure due to intravascular volume loss and rhabdomyolysis with myoglobinuric renal failure, and adequate treatment is required to prevent complications. Treatment involves surgical decompression and rehydration to restore fluid deficits and induce diuresis [1].

Exercise-induced CS has been described in the hand, forearm, leg and thigh, generally developing 24 to 48 hours after exercise [2-5]. Acute CS of the paravertebral muscles without external trauma has rarely been reported.

Lumbar paravertebral CS was first described in a 1985 case report of a young man with postexertional back pain [6]. The diagnosis was based on elevated levels of myoglobin and creatine phosphokinase (CPK), and computed tomography findings suggestive of ischemia and necrosis. Based on the results of a cadaveric dissection and a clinical study of pressure measurements in healthy volunteers, the authors who presented that case reported that the paravertebral muscles were ensheathed in a fascial envelope, which is anatomically and physiologically similar to other muscle compartments known to be susceptible to CS. The compartment is enclosed by the thoracolumbar fascia on the anterior, posterior and lateral sides; and by the spinous processes, interspinous ligaments and attachments of the thoracolumbar fascia on the medial side [7]. The paravertebral compartment pressure has been shown to be affected by posture, the Valsalva maneuver, and trunk extension effort and time. Three etiologies for CS have been identified in the literature [3]. Cases of lumbar paraspinal CS resulting from injury, non-spinal surgery and non-traumatic causes have been reported [8].

## Case presentation

A 30-year-old amateur rugby player participated in weightlifting training mainly targeting the muscles of the lumbar spine. An hour after the end of training, he started to feel left-sided lumbar pain, which gradually developed into severe back pain. The patient did not take regular medications, was a non-smoker and denied using anabolic or illegal substances. He denied any trauma, major impacts during recent rugby games, or previous back pain. Five hours after the end of training, he presented to the emergency room of another hospital and received non-steroidal anti-inflammatory drug treatment. As his vital signs were unremarkable and no neurological symptoms were observed, he was discharged from hospital.

**Figure 1:**
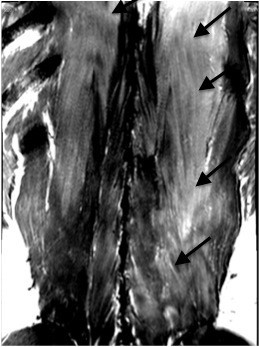
Magnetic resonance imaging (T1-weighted with gadolinium) showing edema and high signal intensity (arrows) in the region of the left multifidus and longissimus muscles.

His pain worsened after discharge, and he re-presented to the hospital a few hours later. As he had severe pain unresponsive to non-steroidal anti-inflammatory drug treatment, he was admitted to the ward for further investigation and intravenous therapy including opioids. Physical examination revealed tenderness to palpation over the lumbar muscles and altered sensation over a 10 × 15 cm area to the left of the lumbar spine. There was no abdominal tenderness, and the remainder of the physical examination was unremarkable. At 24 hours after the end of training, laboratory testing showed a high serum CPK level of 33 000 U/l (normal range 38–174 U/l). Magnetic resonance imaging (MRI) of the lumbar spine showed edema of the multifidus and longissimus muscles (Figure 1). CS was diagnosed and the patient was managed with conservative treatment (administration of pain medication and large volumes of fluids) and close monitoring of renal function including blood gas analysis. Pain medication included hydromorphone, piritramide, diclofenac and paracetamol. The patient continued to experience severe pain, his serum CPK level increased to 43 000 U/l and he had significant myoglobinuria. Repeat MRI at 70 hours after training showed progression of the paravertebral muscle edema and reduced uptake of gadolinium in the left multifidus muscle, indicating decreased perfusion and muscle necrosis. He was referred to the plastic surgery department.

**Figure 2:**
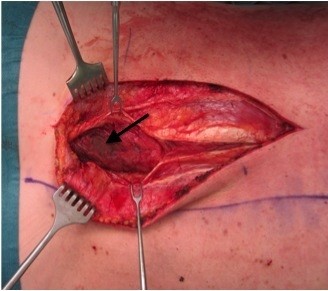
Ischemic non-contractile muscle (arrow).

**Figure 3:**
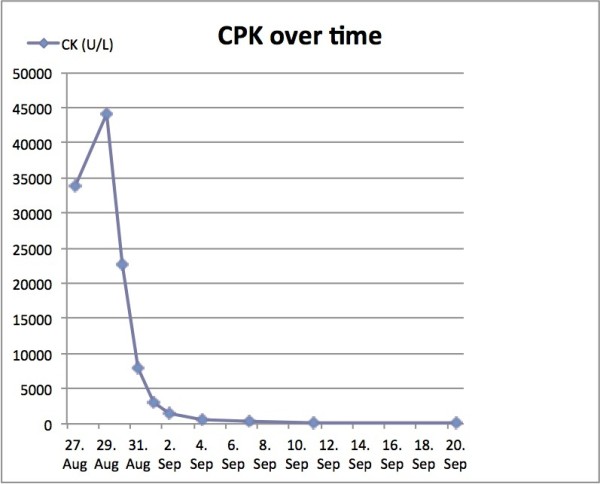
Creatine phosphokinase (CPK) over time.

**Figure 4:**
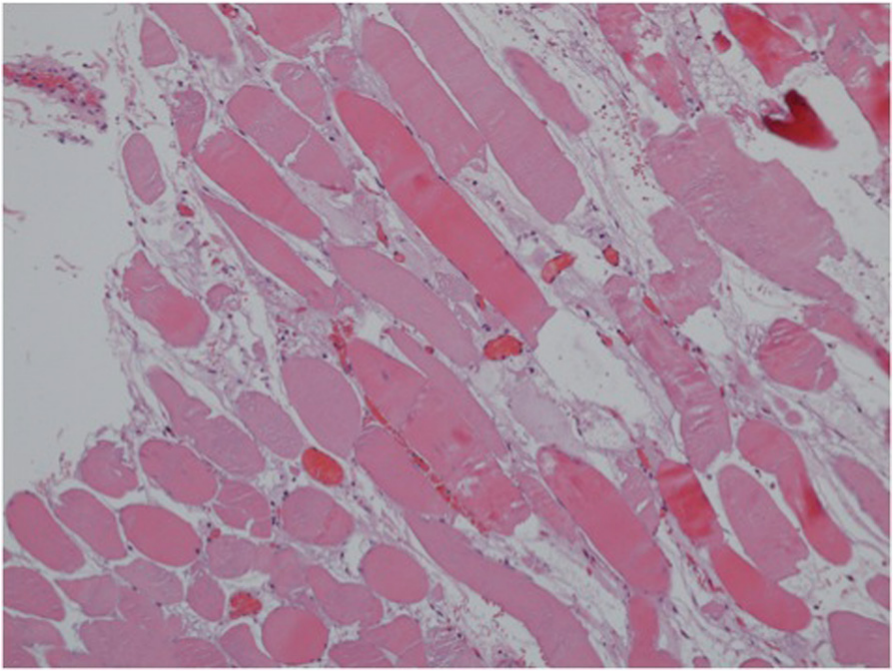
Necrosis of the multifidus muscle with increased interstitial edema (hematoxylin and eosin staining, original magnification × 100).

**Figure 5:**
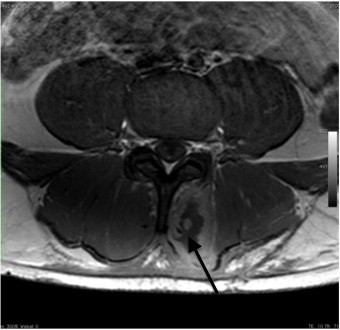
Magnetic resonance imaging (T1-weighted with gadolinium) showing cystic changes (arrow) in the multifidus muscle at 4 weeks after the operation.

Lumbar paravertebral compartment pressure measurements in the prone position (Stryker, Kalamazoo, Michigan) revealed pressures of 3–10 mmHg on the right side and up to 47 mmHg on the left side. The patient’s blood pressure was 110/70 mmHg. Elevation of the left leg in the supine position resulted in increased back pain and was interpreted as a positive stretch sign. The left leg had normal muscle function and no sensory deficits. At 72 hours after the training session, surgical decompression was performed to prevent further muscle necrosis. Under general anesthesia, a left paravertebral incision was made using the Wiltse approach [9]. The thoracolumbar fascia was opened along the plane between the multifidus and longissimus muscles, revealing gray non-contractile muscle tissue (Figure 2). There were no signs of intramuscular hematoma. Muscle necrosis was suspected and a biopsy was taken. The thoracolumbar fascia was left open and the skin incision was closed without tension. Two hours after the operation, the patient’s condition improved significantly. The next morning, he was able to walk and was no longer in severe pain. There was no need for postoperative opioid therapy, and his serum CPK level steadily decreased (Figure 3). The myoglobinuria resolved with continued intravenous hydration. Wound healing was unremarkable. Histopathological examination of the muscle biopsy specimen confirmed necrosis of the multifidus muscle with increased interstitial edema (Figure 4). The patient made a full recovery except for persistence of some sensory deficit, and was discharged from hospital on day 5 after the operation. He regained full, painless range of motion of the spine. Two weeks after the operation, he returned to office work with no further need for pain medication. The Oswestry Disability Index (ODI) was used to document his clinical improvement. The ODI improved from 72 (out of 8 questions, excluding questions about sex life and travelling) on the day of the operation to 18 (out of ten questions) at 3 weeks after the operation. MRI at 4 weeks after the operation showed resolution of the longissimus and iliocostal muscle edema (Figure 5). Hyperintense areas were observed in the multifidus muscle on T2-weighted sequences, with cystic changes that were thought to have resulted from muscle necrosis. At this time, the patient had already resumed light training sessions. He returned to alpine ski sport and rugby training 3 months after the operation, with no signs of any remaining problems, except for mildly decreased sensation over the paravertebral region. Ten months after the operation, the ODI was 2. The patient continues to participate in rugby competitions.

## Conclusions

Based on the results of studies of peripheral CS, various pressures have been suggested as critical limits for surgical intervention. Whitesides et al. proposed that fasciotomy should be performed when the compartment pressure has increased to within 10–30 mmHg of diastolic pressure, or approximately 50–70 mmHg in a normotensive individual [10]. Mubarak and Hargens suggested that an absolute pressure of 30 mmHg was critical [11,12]. Matsen et al. reported that only patients with an intracompartmental pressure of > 45 mmHg could be definitively diagnosed with CS [13]. According to Mubarak, operative decompression is the only satisfactory treatment for CS in the extremities.

Songcharoen studied 20 volunteers to evaluate normal compartment pressures at rest and during weight-lifting exercise [14]. They reported that the mean resting paravertebral compartment pressure was 3.11 mmHg (range 0–11 mmHg) in the prone position and 7.95 mmHg (range 2–20 mmHg) in the sitting position. When a weight of 40 pounds was lifted with a straight back at lift-off, the mean paravertebral compartment pressure was 7.11 mmHg (range 3–30 mmHg), and the pressure returned to the pre-exercise level at 2.16 min (range 1-5 min) after completing the exercise. Peck et al. performed a similar study and reported a mean resting pressure of 10.8 mmHg (7.2-16.4 mmHg) with a mean peak dynamic pressure of 95 mmHg [7]. In all cases, the pressure returned to the normal resting level within 2 seconds of completing the exercise.

Konno et al. studied the relationships between intramuscular pressure of the lumbar back muscles, and pain and degenerative spine disease, in 102 patients with lower back pain with or without neurological deficits [15]. Intramuscular pressure measurements of the lumbar back muscles were performed in various positions. They found that lower back pain was associated with increased intramuscular pressure in the lumbar compartment, and concluded that measurement of intramuscular pressure is an objective method of differentiating between organic and psychogenic back pain. Styf and Lysell reported a patient with exercise-induced chronic back pain caused by chronic CS. They concluded that CS was an uncommon cause of exercise-induced lower back pain [16].

Paravertebral CS has previously been reported in downhill skiers, surfboarders and weight lifters, as well as in reperfusion injury after surgery of the abdominal aorta [6,15,17-21]. Sport-induced CS mainly affects males aged between 20 and 30 years, and generally starts about 2 hours after training. The pain is typically unresponsive to non-narcotic pain medication.

The diagnosis of CS is confirmed by detection of high levels of myoglobin in the serum and urine, a high serum CPK level, and computed tomography findings suggestive of ischemia and necrosis. In all reported cases of paravertebral CS, the main symptom was steadily increasing pain which started within 12 hours of exercise. None of the patients had radicular irritation in the lower extremity. Paravertebral loss of sensation has previously been reported to be a potentially useful sign for early detection of CS. The loss of sensation is thought to result from dysfunction of the lateral branches of the posterior primary rami at multiple levels [22]. Our patient also had a loss of sensation over the paravertebral region. Interestingly, bowel sounds were reported to be diminished in all other patients, with or without abdominal tenderness. Absence of bowel sounds may result from biochemical abnormalities associated with rhabdomyolysis. Our patient had no abdominal symptoms and had normal bowel sounds.

Laboratory findings showed a high serum CPK level and high serum and urine myoglobin levels. Rhabdomyolysis is suspected when the serum CPK level is > 5000 U/l and there are high levels of myoglobin in the serum and urine. In our patient, CS of the paravertebral muscles was indicated by these abnormal laboratory values.

MRI is the preferred radiological examination in patients with suspected CS. Typical MRI findings include muscle edema and hyperintense areas on T2-weighted images. Decreased gadolinium uptake suggests muscle necrosis. In our patient, T2-weighted MRI findings suggested paravertebral muscle edema.

Most previously reported patients with lumbar paravertebral CS were managed by conservative treatment. Careful attention to pain control is very important. Conservative treatment includes administration of analgesics and crystalloid fluid, urine alkalinization and bed rest. In our patient, conservative treatment was not feasible because of the severe pain and increasing serum CPK level. Patients who were managed conservatively experienced only mild back pain on vigorous exertion, and the sequelae of such a condition may be unimportant, making operative decompression unnecessary. Nevertheless, Styf and Lysell reported that fasciotomy relieved the pain in a patient with chronic unilateral lumbar paravertebral CS [16].

In our case, surgery was performed using the Wiltse approach. Wiltse described a modified transmuscular paravertebral approach consisting of longitudinal separation of the sacrospinalis muscle between its multifidus and longissimus parts [9].

To date, only 11 cases of acute paravertebral CS have been reported. Three of these were treated with fasciotomy, resulting in full recovery. We therefore conclude that surgical therapy has good outcomes for both acute and chronic CS.

Two further points should be discussed regarding this case. First, the fascia was left open, but the skin was closed. The decision to close the skin was based on the lack of noticeable tension at the wound margins. Second, none of the gray, non-contractile muscle tissue was removed, except for the biopsy specimen. There was intraoperative discussion regarding the need for total resection of the abnormal muscle tissue. In our opinion, the potential for regeneration of the muscle cannot be determined at the time of decompression, and we therefore did not resect the muscle. Intramuscular scar tissue was assumed to be more beneficial for functional regeneration of this long muscle bundle than the potential muscle defect caused by resection.

In conclusion, acute paravertebral CS should be considered in the differential diagnosis of patients with acute exertional back pain, especially if the pain seems out of proportion to the exertion. We experienced good results after performing surgical decompression in a patient with acute lumbar paravertebral CS after excessive training.

### Consent

Written informed consent was obtained from the patient for publication of this Case report and any accompanying images. A copy of the written consent is available for review by the Editor of this journal.

## Abbreviations

CS: Compartment syndrome; CPK: Creatine phosphokinase; MRI: Magnetic resonance imaging; ODI: Oswestry disability index.

## Competing interests

The authors declare that they have no competing interests.

## Authors’ contributions

GM drafted the manuscript. LL made substantial contributions to the conception of the manuscript. ML, ET, GW and HS gave final approval of the version to be published. All authors read and approved the final manuscript.

## Pre-publication history

The pre-publication history for this paper can be accessed here:

http://www.biomedcentral.com/1471-2474/14/259/prepub
